# An information ecology approach to preferences for online medical consultation platforms in China: a discrete choice experiment

**DOI:** 10.3389/fpubh.2026.1948889

**Published:** 2026-10-29

**Authors:** Li Sun, Dakun Xu

**Affiliations:** School of Public Health, Jilin University, Jilin, China

**Keywords:** China, discrete choice experiment, information ecology, online medical consultation, preference, willingness to pay

## Abstract

**Objectives:**

Online medical consultation (OMC) platforms have become a critical component of digital public health infrastructure in China, yet their sustainability and equitable accessibility are constrained by limited user willingness to pay (WTP). This study aims to quantify preferences and relative WTP for OMC attributes within an information ecology framework, and to examine how these preferences vary across user characteristics and disease contexts.

**Methods:**

A discrete choice experiment with seven attributes was administered via an online survey. Mixed logit models were estimated to derive attribute utilities and relative marginal WTP, with moderation effects and subgroup heterogeneity systematically examined.

**Results:**

A total of 417 valid responses were collected from Chinese adults. Among respondents, social recommendation and physician-generated articles were the most influential attributes, followed by reply count and network environment, while physician rating was not significant. Disease severity was the strongest source of preference heterogeneity: WTP for articles and social recommendation increased substantially from mild to severe conditions. Trust significantly attenuated reliance on external signals, while mobile health literacy reduced price sensitivity. Prior OMC experience consistently elevated relative WTP across most attributes.

**Conclusion:**

This study supports the applicability of information ecology theory to OMC payment decisions in this sample. Social recommendation and physician-generated articles were most valued; trust and mobile health literacy served as key moderators; disease severity fundamentally reshaped preferences. These findings suggest that OMC platform design and payment-related strategies may need to be context-sensitive, particularly with respect to disease severity. Future research and policy discussions may need to consider differential support for users with lower literacy.

## 1 Introduction

Digital health — championed by the World Health Organization for its potential to narrow access gaps and improve care efficiency (1) — is driving a fundamental transformation of global healthcare. As a core branch of digital health, online medical consultation (OMC) supports the synchronous or asynchronous communication between physicians and patients (2), optimizing resource allocation and strengthening public health capacity (3, 4). During the COVID-19 pandemic, contactless online consultations mitigated nosocomial infection risks, supporting both epidemic containment and routine care (5). Although the efficacy of OMC is systematically documented (6), its long-term sustainability depends on users’ willingness to pay (WTP), an issue that remains understudied.

In China, OMC expansion is underpinned by mature internet infrastructure (7). Yet despite this promise, adoption has been hampered by persistent user reluctance to pay, raising concerns about service sustainability and equitable access (8). Addressing this requires a clearer understanding of user preferences and trade-offs (5, 9). Such understanding, in turn, demands a research context that allows users’ real-world trade-off behaviors to be observed and quantified. China’s OMC platforms fall into three types: medical institution, internet company, and local government platforms (10). Among these, the internet company category, exemplified by WeDoctor and Haodf, offers the most suitable setting for preference elicitation. Its transparent pricing, service tiering, and wide range of physician options create conditions that align naturally with discrete choice experiments (DCEs) designed to capture users’ actual attribute trade-offs. We therefore selected this platform type for our study.

Prior studies have applied DCEs to investigate preferences for OMC services, confirming the method’s capacity to quantify attribute importance and WTP (11–13). These studies have identified multidimensional determinants, including physician credentials, consultation fees, and waiting time (5, 9, 11, 14), establishing a solid foundation for understanding user decision-making in this domain. While a few have begun to explore platform-specific ecological cues such as online ratings and physician reputation (15, 16), most DCEs have remained within this traditional clinical domain, leaving broader ecological dimensions relatively underexplored.

Complementing these DCE findings, behavioral theories — including the Technology Acceptance Model, the Unified Theory of Acceptance and Use of Technology, and the Health Belief Model — have explained OMC adoption through individual cognitive perceptions such as perceived usefulness and perceived ease of use (17–20). These frameworks offer valuable insights into technology acceptance but were not designed to capture the multi-attribute trade-offs inherent in out-of-pocket payment decisions. Specifically, they conceptualize the information environment as a passive backdrop, whereas OMC decisions are embedded in an interactive information ecosystem in which user choices are shaped by physician-generated responses (21, 22), peer influence (23), and privacy governance (24). Nor do they address how preference structures may vary by disease severity, as prior heterogeneity analyses have predominantly relied on chronic/non-chronic dichotomies that obscure nuanced variations between mild and severe conditions.

To address these uncharted dimensions, we anchor our investigation in information ecology theory (25). This framework conceptualizes the digital health environment as three interdependent layers — information actors, information, and information environment — that collectively shape how users assess credibility, value, and risk when deciding whether to pay. These layers interact through a compensatory mechanism: when users cannot directly verify a physician’s competence, environmental cues such as social recommendation and network stability serve as credibility proxies that reduce information asymmetry. This logic predicts that internal psychological states such as trust should attenuate reliance on these cues. User characteristics may further moderate how these dimensions influence payment decisions.

By operationalizing these layers through a DCE, this study extends the current evidence in two ways. First, it broadens the attribute scope beyond traditional healthcare dimensions to incorporate information ecology factors (e.g., physician-generated content, social influence, and network environment) that are particularly salient in internet company platform contexts. Second, to test these mechanisms, it moves beyond average effects to examine how users’ psychological characteristics — such as trust and mobile health (mHealth) literacy — moderate attribute evaluations, and explores heterogeneous decision-making patterns across user groups and health conditions. By bridging DCE methodology with information ecology theory, this study offers a framework for understanding digital health payment decisions that is both analytically rigorous and ecologically grounded.

## 2 Methods

### 2.1 DCE methodology

We employed a discrete choice experiment (DCE) grounded in random utility theory to elicit OMC service preferences. The design followed four steps: ① attribute and level identification via literature review informed by information ecology theory; ②orthogonal fractional factorial generation of profiles under mild versus severe disease scenarios; ③ pairwise assembly of choice sets without a no-choice option to encourage active trade-off evaluation; and ④ pilot testing with wording refinements to ensure task clarity.

A forced-choice format was employed, consistent with standard practice in health preference research for estimating attribute trade-offs and marginal WTP. Respondents were required to choose between two hypothetical alternatives, reflecting the real-world constraint that users must often choose between available service options rather than abstaining entirely. Empirical comparisons of forced and unforced choices have found that the relative importance of attributes is generally consistent across both formats (26).

### 2.2 Development of attributes and levels

The selection of attributes and levels is foundational to any DCE, as it determines the validity of preference estimates (27). To ensure comprehensive coverage of factors shaping OMC payment decisions, we grounded our attribute selection in the information ecology framework (25, 28) detailed in the Introduction. Guided by this structure and a systematic literature review, we identified seven candidate attributes, each mapped to one of the three dimensions — information actors, information, and information environment — as detailed below.

#### 2.2.1 Information actors

Information actors — particularly physicians — play a central role in OMC services. Their characteristics, including hospital grade and professional titles, have been shown to significantly shape users’ preferences and willingness to pay in both offline and general online healthcare settings (5, 8). However, on internet-company platforms such as Haodf, physician credentials are structurally homogeneous: tertiary hospitals and chief physicians overwhelmingly dominate the physician pool (15). This homogeneity means credential attributes lack sufficient variation for DCE estimation. The user’s decision problem is therefore not “whether to choose a credentialed physician,” but “how to differentiate among already-credentialed physicians.” Our study focuses on this latter stage, where platform-native signals—article count, historical reputation, reply depth, social recommendation, network environment—become the operative cues.

This study draws on two physician-level attributes that better capture the heterogeneity of information actors on such platforms. Physicians’ professional capital (29) — reflected in the number of health articles they publish — signals professional competence and knowledge reserves, thereby reducing decision-making risks associated with information asymmetry and enhancing users’ willingness to pay. Physicians’ historical reputation (15), as captured by patient-generated rating scores (16), aggregates prior service evaluations and represents the extent to which users recognize the physician’s services, positively shaping users’ expectations of service quality and reinforcing their willingness to pay.

#### 2.2.2 Information

Drawing on prior studies that validated the role of response speed in shaping preferences for online health services (5, 8), and following the operationalization of interaction quality as the frequency of physician-patient interactions in online health communities (30), we included response speed and reply count as indicators of the information dimension. Higher response efficiency enables users to access health guidance more promptly, enhancing service experience and payment willingness (22, 31). Superior communication quality — reflected in more thorough interactions — allows physicians to gain a comprehensive understanding of users’ conditions and provide precise recommendations, thereby strengthening perceived service value and payment willingness.

#### 2.2.3 Information environment

Social influence, network environment, and price constitute the information environment dimension. Social influence, as a third-party reputational signal, effectively reduces decision-making risks; positive social endorsement significantly enhances users’ trust and willingness to pay (22, 32, 33). Network stability ensures smoother service delivery, preventing disruptions that could compromise care continuity and user experience (34). Price operates in the opposite direction: higher prices are associated with lower willingness to pay (35, 36).

To ensure that levels accurately reflect actual platform characteristics while remaining comparable to existing research, we defined each attribute’s levels using a combination of platform data and literature review. Based on the actual distribution of physician data from Haodf platform and established level definitions in prior mobile health WTP studies, 3–4 levels were determined for each attribute, covering the platform’s realistic value ranges with adequate discriminability. The final attribute levels are presented in Table 1.

**Table 1:** Attributes and levels in the discrete choice experiment.

Dimension	Attribute	Levels
Information actor	Article count	2, 15, 50 articles
	Physician rating	4.0points or below; 4.0 + points
Information	Response speed	10, 30, 60 min
	Reply count	3, 8 times
Information environment	Social influence	Not recommended; Neutral; Recommended
	Network environment	Poor; Moderate; Good
	Price (CNY)	¥0; ¥30; ¥80; ¥200

### 2.3 DCE design

We employed an orthogonal design (IBM SPSS 24.0) to generate choice sets from the attribute levels. This approach was chosen because our study is exploratory in nature, aiming to identify broad preference patterns and attribute salience in the absence of reliable prior information on coefficient magnitudes. While D-efficient designs can achieve higher statistical efficiency when accurate priors are available (37), their performance critically depends on the accuracy of assumed priors; misspecification can lead to less robust estimates and may increase respondent cognitive burden (38). Empirical evidence suggests that orthogonal and efficient designs can yield statistically comparable results in certain contexts (39), making the orthogonal approach a balanced and computationally efficient strategy for our study.

The orthogonal design generated 32 profiles, which were paired into 16 choice sets. These were evenly divided into two blocks (eight choice sets per version), with one quality control choice set added to each version to identify inattentive respondents. In this quality control set, one alternative dominated the other on all attributes (see Table 2). The final experimental design thus comprised nine choice sets per version.

**Table 2:** Example of a choice set (quality control question).

Attributes	Option 1	Option 2
Article count	50 articles	2 articles
Physician rating	4.0 + points	4.0points or below
Response speed	10 min	60 min
Reply count	8 times	3 times
Social influence	Recommended	Not recommended
Network environment	Good	Poor
Price (CNY)	¥0	¥200
Your choice	□	□

Respondents were randomly assigned to one of two disease scenarios — mild (symptoms not affecting daily life) or severe (serious illness severely disrupting daily life), with approximately half of the sample assigned to each condition — to examine whether disease severity moderates attribute preferences.

To assess design efficiency, we calculated the D-error under the zero prior assumption. The efficiency calculation was based on a multinomial logit specification for main effects, with attributes coded using effect coding; the quality control choice set was excluded. The final design yielded a D-error of 0.994 (D-efficiency = 1.006), indicating acceptable statistical efficiency for an orthogonal design in health preference studies.

### 2.4 Questionnaire design

The questionnaire consisted of three sections: basic demographic information (gender, age, education level, monthly income, health status, and OMC usage history), discrete choice tasks, and individual characteristics (trust and mHealth literacy).

Trust in OMC platforms was measured using three items adapted from McKnight et al. (40). The original items were contextualized to capture users’ perceptions of privacy/security, service effectiveness, and professional competence in the mobile health setting (40, 41). The specific items are as follows:

1. I believe that current healthcare knowledge platforms can protect my privacy and financial security.
2. I believe that the physicians and knowledge products on these platforms can help me address my health concerns.
3. I believe that the professionalism of physicians and content creators on healthcare knowledge platforms can be guaranteed.

MHealth literacy was measured using items adapted from the Problem-Based mHealth Literacy Scale developed by Zhang and Li (42), which captures individuals’ perceived abilities in acquiring, evaluating, and applying mobile health information for health-related decision-making. The specific items are as follows:

1. I know which online platforms I can access for useful health and medical information.
2. I know how to use the health information I obtain online to help myself.
3. I have the cognitive ability to judge the quality of online health and medical information.
4. I feel confident in my ability to make health-related decisions using online information.

Both scales employed a 5-point Likert scale (see Supplementary Material for details).

Reliability and validity testing showed that Cronbach’s α coefficients for the trust and mHealth literacy scale were 0.81 and 0.80, respectively; all standardized factor loadings exceeded 0.7; average variance extracted (AVE) values were 0.66 and 0.57; and composite reliability (CR) values were 0.85 and 0.84. These results indicate strong internal consistency and convergent validity, confirming the suitability of both scales for subsequent analyses.

Besides, this study was approved by the Ethics Committee of the School of Public Health, Jilin University (Approval code: 2025-01-03, 10 January 2025).

### 2.5 Data collection

Following the rule of thumb established by Johnson and Orme (43, 44), the minimum sample size for a discrete choice experiment should satisfy N > 500c/(t × a), where c is the largest number of levels across any attribute, t is the number of DCE choice tasks per respondent, and a is the number of alternatives per choice task. Based on this formula, the minimum required sample size was calculated as 500 × 4 / (8 × 2) = 125. In addition, prior research also suggests that larger samples help mitigate statistical bias (45). Taken together, the target effective sample size for this study was set at 300.

A pilot test with 20 participants was conducted to assess the reliability and validity of the questionnaire and to refine the wording of the items. Data were collected through an online survey administered via Wenjuanxing (www.wjx.cn) between July and October 2025. Due to the open distribution and voluntary participation nature of the study, convenience sampling combined with snowball sampling was employed. The survey link was distributed through the platform’s public participant pool and social media channels, and initial respondents were encouraged to share it with peers. This recruitment strategy was pragmatic, given the exploratory nature of the study and the difficulty of accessing a probability-based sample of OMC users in China. Convenience sampling is widely recognized as appropriate for exploratory behavioral research (46), and snowball sampling helps expand respondent reach within social networks (47). While these non-probability techniques limit full generalizability, they are increasingly used in digital survey environments where fully randomized samples are often infeasible (48).

Following pre-specified quality criteria, 194 respondents (27.06%) failed the attention check and 106 (14.78%) exhibited straight-lining; both were excluded. A total of 417 valid responses were obtained (overall exclusion rate: 41.84%), exceeding the target sample size. To assess potential systematic bias, we re-estimated the mixed logit model on both the full sample (N = 717) and the valid sample (N = 417) (see Supplementary Table S1). The coefficient signs and significance levels of all core variables remained consistent, indicating that the exclusion criteria did not systematically affect the results.

## 3 Statistical analysis

### 3.1 Discrete choice model specification

The mixed logit model was employed as the primary analytical framework. Relative to the standard multinomial logit model, it allows coefficients to follow specified distributions (e.g., normal, log-normal), thereby capturing preference heterogeneity across individuals — a feature critical to OMC service selection contexts. The specification assumes a linear utility for the price attribute and holds the price coefficient constant, consistent with standard practice in applied choice modelling (49, 50). We tested the linearity assumption using two complementary approaches: re-estimating the model with a quadratic price term, and treating price as a categorical variable. Both tests indicated non-linearity. We retained the linear specification for the main analysis for three reasons: ① it yields WTP estimates directly from the distributions of non-price coefficients, whereas non-linear price specifications require defining marginal price utility at a reference price point, which complicates both computation and interpretation; ② the present study focuses on the drivers of WTP and their heterogeneity across respondents rather than on the shape of price sensitivity, and the non-linear specification would substantially complicate the subsequent interaction and heterogeneity analyses; and ③ the core findings were robust across specifications. Results of the non-linearity tests are reported in Supplementary Tables S2, S3.

### 3.2 Willingness-to-pay calculation

Marginal WTP for each non-price attribute was derived as the negative ratio of the attribute coefficient to the price coefficient. Positive values indicate willingness to pay for attribute improvements, while negative values imply required compensation.

### 3.3 Moderation effect testing

To examine the moderating roles of trust and mHealth literacy, interaction terms were introduced into the model. Specifically, trust was interacted with social influence and network environment to verify whether trust attenuates users’ reliance on external signals, and mHealth literacy was interacted with article count and price to assess whether users’ level of mHealth literacy affects their valuation of information richness and their sensitivity to price. Prior to model estimation, trust and mHealth literacy were mean-centered to control for potential multicollinearity. Significant interaction coefficients indicate moderating effects.

### 3.4 Heterogeneity analysis

Subgroup analyses were conducted by demographic characteristics, OMC experience and disease contexts. Models were estimated separately for each subgroup, with likelihood ratio tests used to assess whether coefficient differences across groups were statistically significant. To examine preference heterogeneity, we further estimated a two-class latent class logit model (LCLM). Given the limited sample size (N = 417), we used a parsimonious specification with three core attributes: price, article count, and social influence. The model was estimated using the lclogit command in Stata with default settings. As a robustness check, we also estimated a four-attribute model including reply count. Model fit was compared using AIC, BIC, and log-likelihood values, and the stability of class assignment was assessed by the agreement rate between the two models.

## 4 Results

### 4.1 Descriptive statistical results

A total of 417 valid responses were collected, with descriptive statistics presented in Table 3. The sample comprised 71.2% female respondents, predominantly aged 18–40 years (71.7%), holding a bachelor’s degree or above (81.3%), and reporting monthly incomes between RMB 1,000 and 5,000 (65.4%). Prior OMC experience was reported by 85.6% of respondents. The distribution across mild and severe disease scenarios was relatively balanced (44.4% vs. 55.6%). For subsequent analyses, respondents aged 56 years and above were combined with the 41–55 age group into a single category of 41 years and above (n = 118, 28.3%), and the self-rated health categories of “very poor” and “poor” were merged into a “poor” group (n = 25, 6.0%).

**Table 3:** Sample descriptive statistics (*N* = 417).

Characteristic	Category	Frequency	Percentage (%)
Gender	Male	120	28.8
	Female	297	71.2
Age	18–25 years	175	42.0
	26–40 years	124	29.7
	41–55 years	98	23.5
	56 years and above	20	4.8
Education	High school or below	45	10.8
	Junior college	33	7.9
	Bachelor’s degree	247	59.2
	Master’s degree or above	92	22.1
Monthly income (RMB)	< 1,000	30	7.2
	1,000–3,000	184	44.1
	3,001–5,000	89	21.3
	5,001–10,000	80	19.2
	> 10,000	34	8.2
Self-rated health	Very good	111	26.6
	Fair	281	67.4
	Poor	23	5.5
	Very poor	2	0.5
OMC experience	Yes	357	85.6
	No	60	14.4
Disease scenario	Mild condition	185	44.4
	Severe condition	232	55.6

### 4.2 Main effects: preferences and WTP (baseline model)

The mixed logit model estimation results are presented in Table 4. The model showed good fit (Log likelihood = −2,008; LR chi-square = 255, *p* < 0.001), indicating that the included attributes jointly explain users’ preferences for OMC services.

**Table 4:** Mixed logit model results.

Attribute	Level	*β*	*SE*	*p*-value	WTP (CNY)
Information actor					
Article count	2 articles (ref.)	—	—	—	—
	15 articles	0.737***	0.149	<0.001	66.97
	50 articles	0.889***	0.150	<0.001	80.79
Physician rating	4.0 or below (ref.)	—	—	—	—
	4.0 + points	0.156	0.115	0.173	14.19
Information					
Response speed	60 min (ref.)	—	—	—	—
	30 min	−0.625***	0.187	0.001	−56.83
	10 min	−0.202	0.141	0.154	−18.33
Reply count	3 times (ref.)	—	—	—	—
	8 times	0.452***	0.082	<0.001	41.11
Information environment					
Social influence	Not recommended (ref.)	—	—	—	—
	Neutral	−0.110	0.179	0.540	−9.98
	Recommended	0.743***	0.106	<0.001	67.50
Network environment	Poor (ref.)	—	—	—	—
	Moderate	0.135	0.112	0.227	12.28
	Good	0.572**	0.199	0.004	51.97
Price	—	−0.011***	0.001	<0.001	—

Observations = 6,672, AIC = 4,043, BIC = 4,123, Log likelihood = −2,008, LR chi-square = 255 (*p* < 0.001). ***p < 0.001, **p < 0.01. WTP was calculated as the negative ratio of the attribute coefficient to the price coefficient.

Among the information actor attributes, the number of articles exhibited a significant positive effect. Relative to the reference level of 2 articles, publishing 15 articles (*β* = 0.737, *p* < 0.001) and 50 articles (*β* = 0.889, *p* < 0.001) significantly increased utility, corresponding to WTPs of ¥66.97 and ¥80.79, respectively. Physician rating, — though widely emphasized in offline settings — did not show a significant effect (*β* = 0.156, *p* = 0.173) in the context of internet company platforms.

For the information dimension, both response speed and reply count demonstrated significant influences. Compared to a 60-min response time, a 30-min wait showed a negative coefficient (*β* = −0.625, *p* = 0.001), with a WTP of -¥56.83—suggesting that this level was perceived as inferior to the reference. The 10-min level, though also negative, was not statistically significant (*β* = −0.202, *p* = 0.154). In contrast, increasing reply count from 3 to 8 times had a positive effect (*β* = 0.452, *p* < 0.001), with a WTP of ¥41.11.

In the information environment dimension, social influence emerged as a strong predictor. A “recommended” status significantly increased utility relative to “not recommended” (*β* = 0.743, *p* < 0.001), with a WTP of ¥67.50. A “neutral” status showed no significant effect. Network environment also mattered: “good” network quality (*β* = 0.572, *p* = 0.004) generated a WTP of ¥51.97, while “moderate” quality was not statistically significant. Price was negatively associated with utility, as expected (*β* = −0.011, *p* < 0.001), confirming that higher costs reduce users’ likelihood of choosing a given service.

### 4.3 Moderation effects of trust and mHealth literacy

After incorporating the moderating effects, the direction and significance of the core attribute preferences remained consistent with the baseline model. Moderating effects results are presented in Table 5 (see Supplementary Table S4 for details).

**Table 5:** Moderating effects results (interaction terms only).

Interaction term	Level	*β*	SE	*p*-value
Trust × social influence	Not recommended (ref.)	—	—	—
	Neutral	−0.053	0.149	0.720
	Recommended	−0.309**	0.098	0.002
Trust × network environment	Poor (ref.)	—	—	—
	Moderate	−0.027	0.104	0.794
	Good	−0.328*	0.167	0.049
mHealth literacy × article count	2 articles (ref.)	—	—	—
	15 articles	−0.086	0.110	0.431
	50 articles	0.153	0.111	0.168
mHealth literacy × price	—	0.002**	0.001	0.009

Main effects are reported in Supplementary Table S4 for details. Only interaction terms are shown here. **p < 0.01,*p < 0.05.

#### 4.3.1 Trust

Trust significantly reduced the marginal utility of social recommendation (*β* = −0.309, *p* = 0.002) and good network environment (*β* = −0.328, *p* = 0.049). This pattern suggests that users with higher trust rely less on external signals (social endorsement or network stability) and are more tolerant of environmental imperfections. No significant moderation was found for neutral social influence or moderate network quality.

#### 4.3.2 mHealth literacy

The interaction between mHealth literacy and article count was not significant (15 articles: *β* = −0.086, *p* = 0.431; 50 articles: *β* = 0.153, *p* = 0.168), indicating that users’ valuation of information richness does not vary with mHealth literacy. However, mHealth literacy significantly attenuated price sensitivity (*β* = 0.002, *p* = 0.009), suggesting that higher-literacy users are less deterred by price increases, likely due to a stronger appreciation of OMC service value.

### 4.4 Subgroup analysis

Tables 6, 7 present the marginal WTP estimates across four selected subgroups. Detailed results of the subgroup analyses are shown in Supplementary Tables S5–S8. Several patterns emerge. First, the WTP for physician-generated content and social recommendation increased substantially with disease severity—from ¥56.27 (mild) to ¥105.01 (severe) for 50 articles (*p* < 0.001), and from ¥38.22 (mild) to ¥110.41 (severe) for social recommendation (*p* < 0.001). These patterns indicate that WTP for informational resources and social confirmation was substantially higher under severe conditions. Second, users with prior OMC experience exhibited consistently higher WTP than first-time users across most attributes, particularly for article count (¥81.11 vs. ¥59.06, *p* < 0.001) and reply count (¥40.59 vs. ¥30.87, *p* < 0.001), indicating that experience may enhance perceived service value. Third, males showed higher WTP than females for most attributes, with the largest gap observed for 50 articles (¥122.33 vs. ¥66.10, *p* < 0.001). Fourth, age-related differences were most pronounced for social influence: the ≥41 group exhibited the highest WTP for social recommendation (¥108.61, *p* < 0.001), suggesting that older users rely more heavily on social signals when making OMC decisions.

**Table 6:** Marginal WTP estimates by demographic characteristics (CNY).

Attribute	Level	Gender		Age		
		Male	Female	18–25	26–40	≥41
Information actor						
Article count	2 articles (ref.)	—	—	—	—	—
	15 articles	99.99	54.09**	80.38***	58.87*	47.52
	50 articles	122.33***	66.10***	89.19***	89.29***	49.90
Physician rating	4.0 or below (ref.)	—	—	—	—	—
	>4.0 points	44.63	4.11	28.90	14.20	−12.82
Information						
Response speed	60 min (ref.)	—	—	—	—	—
	30 min	−120.09**	−29.67	−62.27*	−52.56	−31.46
	10 min	−42.94	−6.46	−13.29	−21.18	−2.23
Reply count	3 times (ref.)	—	—	—	—	—
	8 times	73.99***	29.31***	44.13***	41.65**	37.74*
Information environment						
Social influence	Not recommended (ref.)	—	—	—	—	—
	Neutral	−47.39	8.30	−10.02	1.42	−0.56
	Recommended	73.52**	64.74***	55.18***	46.50**	108.61***
Network environment	Poor (ref.)	—	—	—	—	—
	Moderate	17.14	14.21	14.10	14.03	16.41
	Good	23.53	66.74**	47.02	64.31*	69.60

WTP was calculated as the negative ratio of the attribute coefficient to the price coefficient. ***p < 0.001, **p < 0.01,*p < 0.05.

**Table 7:** Marginal WTP estimates by OMC experience and disease context (CNY).

Attribute	Level	OMC experience		Disease context	
		Yes	No	Mild	Severe
Information actor					
Article count	2 articles (ref.)	—	—	—	—
	15 articles	66.35***	55.30	56.78**	74.69**
	50 articles	81.11***	59.06	56.27**	105.01***
Physician rating	4.0 or below (ref.)	—	—	—	—
	>4.0 points	20.48	−26.39	9.39	23.28
Information					
Response speed	60 min (ref.)	—	—	—	—
	30 min	−50.62**	−61.22	−96.27***	−5.79
	10 min	−8.62	−59.62	−55.57***	33.05
Reply count	3 times (ref.)	—	—	—	—
	8 times	40.59***	30.87	31.49**	54.70***
Information environment					
Social influence	Not recommended (ref.)	—	—	—	—
	Neutral	−8.07	10.88	−67.52**	56.88*
	Recommended	62.99***	67.66*	38.22**	110.41***
Network environment	Poor (ref.)	—	—	—	—
	Moderate	15.43	0.86	−11.92	45.02**
	Good	44.71*	112.23	−11.55	126.39***

WTP was calculated as the negative ratio of the attribute coefficient to the price coefficient. ***p < 0.001, **p < 0.01, *p < 0.05.

The three-attribute LCLM identified two classes: a price-sensitive class (66.2%, n = 276) and a price-insensitive class (33.8%, n = 141). The price-sensitive class showed a strong negative effect of the 200 CNY level (*β* = −2.792, *p* < 0.001), whereas the price-insensitive class showed no significant price effect (*β* = 0.300, *p* = 0.247). Model fit: log-likelihood = −2035.04, AIC = 4100.09, BIC = 4160.58. The four-attribute model produced a similar structure (price-sensitive: 62.8%; price-insensitive: 37.2%), with 62.11% agreement in class assignment. These findings provide empirical evidence for the presence of preference heterogeneity. Detailed results are in Supplementary Tables S9–S11.

## 5 Discussion

This study applied the information ecology framework to investigate public preferences and WTP for OMC services via a DCE. The findings offer theoretical and practical insights.

### 5.1 Core findings

The baseline model revealed a clear preference hierarchy. Article count was the most influential attribute: users were willing to pay ¥80.79 for 50 articles (versus the 2-article baseline), followed by social recommendation (¥67.50), network environment (¥51.97), and reply count (¥41.11). When interpreted through the information ecology framework, these findings reveal distinct patterns across its three constitutive dimensions.

From the information actor dimension, article count emerged as the most valued attribute. This finding—not previously reported in OMC DCE studies—suggests that physician-generated content serves as a credible signal of professional competence and knowledge reserves. This extends prior research that focused primarily on structured signals such as titles and hospital tiers (5, 35), highlighting the importance of “information actor” characteristics in shaping user preferences. In contrast, physician rating was not significant in our model, diverging from prior DCE findings in China where rating consistently dominated preferences (5). This non-significance may reflect the specific context of internet company platforms, where users have access to richer informational cues—articles, reply quality, and social signals—that supplement or substitute for ratings. When users can observe a physician’s knowledge output and interaction style directly, the marginal value of a summary rating diminishes.

From the information dimension, reply count significantly drove WTP (¥41.11), indicating that users value interaction depth as a proxy for service thoroughness. Evidence from online health communities confirms that interaction quality — operationalized as the frequency and depth of physician-patient exchanges — significantly influences patients’ adoption decisions (51). This suggests that users do not merely seek answers; they seek evidence of the physician’s engagement and attentiveness through the communication process. The number of replies serves as a visible and quantifiable signal of the physician’s willingness to invest time and cognitive effort, thereby reducing uncertainty about service quality. Conversely, response speed (30 min) showed a significant negative coefficient (WTP: –¥56.83), suggesting that, on average, faster replies were perceived as inferior to the 60-min baseline. However, this effect did not remain significant when price non-linearity was accounted for, indicating that the significance of this attribute in the baseline model may be sensitive to the price specification. Importantly, the direction reversal across disease contexts — the focus of Section 5.3 — remained robust to alternative price specifications. We therefore focus our interpretation on the heterogeneity finding rather than the baseline main effect. This counterintuitive pattern underscores that efficiency cues are not uniformly valued and may be interpreted differently depending on the health context.

From the information environment dimension, both social recommendation and network environment proved influential. Social recommendation (¥67.50) ranked second in overall importance, aligning with prior work demonstrating that peer endorsement reduces perceived decision risk in online health choices (52, 53). In the absence of direct experience with a physician, users rely on aggregated social signals as a heuristic for quality assessment — consistent with the social dimension of information ecology, where user-generated signals serve as credible decision-making shortcuts that complement professional credentials. Network environment (¥51.97), while moderately influential overall, showed substantially elevated WTP under severe conditions (¥126.39), underscoring the importance of technical infrastructure — a factor rarely examined in prior DCE research. Systematic reviews have consistently identified technology infrastructure as a key determinant of health technology adoption and implementation (54), yet its role in shaping users’ payment decisions has remained largely unquantified. Our findings quantify users’ WTP for stable networks, revealing that technical infrastructure is valued beyond being a mere service precondition. The price coefficient was negative and statistically significant (*β* = −0.011, *p* < 0.001), consistent with economic theory and confirming that users’ utility decreases as consultation fees increase. This validates the WTP estimates derived from the attribute-to-price coefficient ratios reported above.

Collectively, these findings suggest that, in the Chinese OMC context, users in this sample draw on a broader set of informational and environmental cues — extending well beyond traditional physician credentials — to inform OMC payment decisions. The information ecology framework suggests that value is derived not from any single attribute but from the interplay of information actors, content, and environment. These findings suggest that OMC platform design may need to consider content richness, interaction depth, and network reliability However, the WTP estimates should be interpreted as relative preferences rather than direct guidance for pricing or coverage decisions. At the policy level, these findings raise questions about whether technical infrastructure and content quality should be considered in platform governance, although this remains a hypothesis for future research.

### 5.2 Moderating roles of trust and mHealth literacy

Beyond the main effects of platform attributes, we examined whether users’ psychological characteristics—trust and mHealth literacy—moderate the relationships between specific attributes and WTP. The information ecology framework does not merely classify attributes into three categories; it predicts a compensatory mechanism across dimensions. This mechanism posits that when users cannot directly verify a platform physician’s competence, environmental cues serve as credibility proxies, and internal psychological states such as trust should attenuate reliance on these cues.

Consistent with this prediction, trust significantly attenuated the marginal utility of social recommendation (*β* = −0.309, *p* = 0.002) and good network environment (*β* = −0.328, *p* = 0.049), consistent with its cognitive substitution function. Higher-trust users rely less on external signals and tolerate suboptimal conditions better (55). This extends the organizational trust model to digital health and complements recent work showing trust moderates social influence on OMC adoption (53). This aligns with the elaboration likelihood model (ELM) (56), when central cues are available (e.g., physician-generated articles), users rely less on peripheral environmental signals. Conversely, when central cues are weak or absent, environmental signals become the primary basis for value assessment. This substitution dynamic is the core theoretical contribution of applying information ecology to OMC payment decisions.

Concurrently, mHealth literacy significantly attenuated price sensitivity (*β* = 0.002, *p* = 0.009). Higher-literacy users were less deterred by price increases, consistent with national evidence identifying eHealth literacy as the strongest predictor of WTP for OMC in China (57). This literacy–price interaction suggests that value perception is not solely determined by objective service features but is actively constructed through users’ capacity to process health information. Higher-literacy users can better recognize quality signals embedded in platform attributes—such as the informational value of physician articles or the thoroughness implied by reply count—and are therefore more willing to pay for services that deliver these benefits. From a policy perspective, enhancing literacy not only facilitates initial adoption but also strengthens acceptance of reasonable pricing, supporting sustainable platform strategies from the demand side.

In the Chinese OMC context, trust and mobile health literacy emerged as key moderators of payment preferences. These findings suggest that platform design may need to consider how trust is built and maintained, particularly for users with limited prior OMC experience, and that future research should examine whether literacy support tools can improve comprehension and willingness to pay among users with lower mHealth literacy. At the policy level, these findings raise questions about whether mHealth literacy should be considered in efforts to promote equitable access, although this remains a hypothesis for future research.

### 5.3 Heterogeneity analysis

Subgroup analyses were conducted by disease context, OMC experience, gender, and age. The most pronounced heterogeneity emerged from disease severity and prior experience, with more modest variations by gender and age.

Disease severity emerged as the strongest source of preference heterogeneity. Disease severity substantially amplified users’ valuation of both informational and social resources. WTP for 50 articles increased by 86.7% (from ¥56.27 to ¥105.01), while WTP for social recommendation surged by 188.9% (from ¥38.22 to ¥110.41), suggesting that users facing more serious conditions place greater value on informational support and social confirmation. This pattern aligns with the severity hypothesis, which posits that health services yielding a fixed utility gain are valued more highly when the patient’s initial health state is more severe (58). The present study extends this hypothesis to OMC payment decisions, demonstrating that disease severity not only elevates overall WTP but also fundamentally reshapes attribute preference structures — with severe-condition users placing substantially greater weight on informational and social cues. This finding is further corroborated by evidence that perceived disease severity positively predicts health information-seeking behavior (59).

Response speed showed a direction shift across disease contexts. Faster responses (30 min, 10 min) were negatively valued relative to 60 min under mild conditions (WTP: –¥96.27, −¥55.57), whereas the 10-min response was positive but non-significant under severe conditions (WTP: ¥33.05). Re-estimating the subgroup models with a quadratic price term confirmed this pattern (mild: *β* = −0.924, *p* = 0.003; severe: *β* = 3.244, *p* = 0.188). Thus, the direction of the divergence was robust across specifications. The divergence may be understood through signaling theory (60). In mild conditions, a 30-min response may signal “low effort” or “superficial consultation,” leading to negative utility. In severe conditions, the signaling value may flip: a 30-min response may signal “high responsiveness” and “prioritization,” while a 60-min delay may signal “negligence.” This shift may also occur because anxiety reduces the capacity for systematic processing, under which heuristic cues disproportionately shape judgment (61). High anxiety in severe conditions may impair systematic processing and amplify reliance on heuristic cues (e.g., “faster is safer”), whereas low-stakes mild conditions may allow systematic evaluation in which users can afford to be critical of “too fast” services. Together, these two lenses suggest that the meaning of response speed is not fixed but may be actively constructed through the interplay between health context, cognitive–emotional state, and the signal conveyed by the attribute level. However, given that the severe-scenario estimate was non-significant in both linear and quadratic specifications, this interpretation should be treated as a plausible explanation rather than a confirmed effect, and future research with larger or patient samples is needed to test it directly.

Prior OMC experience was another important source of heterogeneity. Prior OMC experience consistently elevated WTP across most attributes, with the largest gaps for article count (¥81.11 vs. ¥59.06, *p* < 0.001) and reply count (¥40.59 vs. ¥30.87, *p* < 0.001). This pattern reveals a digital health divide mechanism: inexperienced users, lacking accurate value assessment, exhibit suppressed WTP. This corroborates prior research (12, 62) showing that prior experience significantly increases WTP for health apps. These findings raise the question of whether literacy support or guided onboarding at the first-use stage can help inexperienced users form more accurate value perceptions. Whether such interventions should target older or lower-education groups remains an open question, as the present sample was skewed toward younger, female, and highly educated users.

Gender and age also showed modest heterogeneity. Males exhibited higher WTP for article count and reply count, while females valued network environment more. Age-related differences were most pronounced for social influence: older users (≥41) demonstrated the highest WTP for social recommendation (¥108.61, *p* < 0.001), suggesting greater reliance on social signals in OMC decisions. These patterns broadly align with prior DCE findings (5, 8).

These findings suggest that future research should examine whether OMC service design and payment-related strategies need to be differentiated by disease context and user experience, rather than assuming uniform attribute value across subgroups.

Across all analyses, the WTP estimates should be interpreted as relative preferences among attribute levels within the DCE design, rather than absolute willingness to pay in real-world markets. Because the choice tasks were hypothetical and forced-choice, these estimates capture attribute trade-offs rather than actual payment behavior.

### 5.4 Limitations

Several limitations warrant mention. First, the sample was recruited through convenience and snowballing sampling via an online platform, which may limit generalizability. The sample skewed toward younger, female, and highly educated individuals, and all respondents were lay users rather than confirmed patients responding to hypothetical disease vignettes. While the substantial differences in WTP estimates between mild and severe scenarios suggest that respondents responded to the scenario manipulation, these findings should be interpreted as stated preferences under hypothetical conditions rather than observed patient behavior. We cannot fully rule out that some respondents may have anchored to their own health circumstances rather than fully adopting the hypothetical scenario. Additionally, findings from internet company platforms may not generalize to other platform types (e.g., medical institution platforms, local government platforms).

Second, to the extent that physician credentials function as non-negotiable screening criteria, their exclusion may have led to an overestimation of WTP for the secondary cues examined in this study (e.g., article count, social recommendation), as respondents who would otherwise prioritize credential attributes may have redirected their valuation toward the platform-native attributes available in the DCE design. The generalizability of our findings is therefore bounded to platform contexts with structurally constrained credential variation.

Third, the DCE employed a forced-choice format without an opt-out option. This may have led to an overestimation of WTP for certain attributes, and future studies consider including an opt-out alternative when the research objective is to predict actual uptake rather than estimate marginal WTP.

Finally, the linear price specification used to calculate WTP is a simplification. Our tests indicate that the price–utility relationship is non-linear, and WTP estimates derived from the linear model may be biased at extreme price levels. Given that the core findings were robust across specifications, we accepted this trade-off to preserve comparability and analytical tractability. Future studies should consider non-linear price specifications.

## Data Availability

The raw data supporting the conclusions of this article will be made available by the authors, without undue reservation.
