# Supplementary material for: An information ecology approach to preferences for online medical consultation platforms in China: a discrete choice experiment
Source: Front Public Health. 2026 Oct 29;14:1948889. doi: 10.3389/fpubh.2026.1948889 (PMC13754543; doi:10.3389/fpubh.2026.1948889)
Supplement: Supplementary file 1 [file Supplementary_file_1.DOCX]

Supplementary Material

# Additional file 1: the DCE questionnaire

### **DCE Choice Sets (Mild Disease Scenario)**

**Instruction:** Suppose you have a mild disease or experience mild symptoms that cause discomfort but do not significantly affect your daily life.

| **Choice Set ① Which option would you prefer? Please tick your choice.** | | |
| --- | --- | --- |
| Attributes | Option 1 | Option 2 |
| Number of articles | 2 articles | 15 articles |
| Physician rating | 4.0 or below | 4.0+ points |
| Response speed | 10 min | 30 min |
| Reply count | 8 times | 3 times |
| Social influence | Neutral | Neutral |
| Network environment | Moderate | Good |
| Price (CNY) | ¥80 | ¥80 |
| **Your choice** | □ | □ |
| **Choice Set ② Which option would you prefer? Please tick your choice.** | | |
| Attributes | Option 1 | Option 2 |
| Number of articles | 50 articles | 15 articles |
| Physician rating | 4.0+ points | 4.0+ points |
| Response speed | 10 min | 30 min |
| Reply count | 3 times | 8 times |
| Social influence | Neutral | Recommended |
| Network environment | Poor | Moderate |
| Price (CNY) | ¥30 | ¥30 |
| **Your choice** | □ | □ |
| **Choice Set ③ Which option would you prefer? Please tick your choice.** | | |
| Attributes | Option 1 | Option 2 |
| Number of articles | 15 articles | 2 articles |
| Physician rating | 4.0+ points | 4.0+ points |
| Response speed | 10 min | 10 min |
| Reply count | 3 times | 8 times |
| Social influence | Not recommended | Recommended |
| Network environment | Moderate | Poor |
| Price (CNY) | ¥0 | ¥0 |
| **Your choice** | □ | □ |
| **Choice Set ④ Which option would you prefer? Please tick your choice.** | | |
| Attributes | Option 1 | Option 2 |
| Number of articles | 15 articles | 2 articles |
| Physician rating | 4.0 or below | 4.0+ points |
| Response speed | 60 min | 60 min |
| Reply count | 3 times | 8 times |
| Social influence | Recommended | Not recommended |
| Network environment | Poor | Poor |
| Price (CNY) | ¥0 | ¥80 |
| **Your choice** | □ | □ |
| **Choice Set ⑤ Which option would you prefer? Please tick your choice.** | | |
| Attributes | Option 1 | Option 2 |
| Number of articles | 50 articles | 2 articles |
| Physician rating | 4.0 or below | 4.0 or below |
| Response speed | 60 min | 10 min |
| Reply count | 8 times | 3 times |
| Social influence | Not recommended | Not recommended |
| Network environment | Good | Poor |
| Price (CNY) | ¥80 | ¥0 |
| **Your choice** | □ | □ |
| **Choice Set ⑥ Which option would you prefer? Please tick your choice.** | | |
| Attributes | Option 1 | Option 2 |
| Number of articles | 2 articles | 2 articles |
| Physician rating | 4.0 or below | 4.0 or below |
| Response speed | 10 min | 30 min |
| Reply count | 8 times | 3 times |
| Social influence | Not recommended | Recommended |
| Network environment | Poor | Poor |
| Price (CNY) | ¥200 | ¥80 |
| **Your choice** | □ | □ |
| **Choice Set ⑦ Which option would you prefer? Please tick your choice.** | | |
| Attributes | Option 1 | Option 2 |
| Number of articles | 2 articles | 2 articles |
| Physician rating | 4.0 or below | 4.0+ points |
| Response speed | 30 min | 10 min |
| Reply count | 8 times | 3 times |
| Social influence | Neutral | Neutral |
| Network environment | Poor | Poor |
| Price (CNY) | ¥30 | ¥200 |
| **Your choice** | □ | □ |
| **Choice Set ⑧ Which option would you prefer? Please tick your choice.** | | |
| Attributes | Option 1 | Option 2 |
| Number of articles | 50 articles | 15 articles |
| Physician rating | 4.0 or below | 4.0+ points |
| Response speed | 10 min | 10 min |
| Reply count | 8 times | 8 times |
| Social influence | Neutral | Not recommended |
| Network environment | Moderate | Good |
| Price (CNY) | ¥0 | ¥200 |
| **Your choice** | □ | □ |
| **Choice Set ⑨ Which option would you prefer? Please tick your choice.** | | |
| Attributes | Option 1 | Option 2 |
| Number of articles | 50 articles | 2 articles |
| Physician rating | 4.0+ points | 4.0 or below |
| Response speed | 10 min | 60 min |
| Reply count | 8 times | 3 times |
| Social influence | Recommended | Not recommended |
| Network environment | Good | Poor |
| Price (CNY) | ¥0 | ¥200 |
| **Your choice** | □ | □ |

### **DCE Choice Sets (Severe Disease Scenario)**

**Instruction:** Suppose you have a severe disease or experience severe symptoms that significantly affect your daily life.

| **Choice Set ① Which option would you prefer? Please tick your choice.** | | |
| --- | --- | --- |
| Attributes | Option 1 | Option 2 |
| Number of articles | 15 articles | 50 articles |
| Physician rating | 4.0 or below | 4.0+ points |
| Response speed | 60 min | 30 min |
| Reply count | 8 times | 3 times |
| Social influence | Neutral | Not recommended |
| Network environment | Poor | Poor |
| Price (CNY) | ¥200 | ¥200 |
| **Your choice** | □ | □ |
| **Choice Set ② Which option would you prefer? Please tick your choice.** | | |
| Attributes | Option 1 | Option 2 |
| Number of articles | 2 articles | 50 articles |
| Physician rating | 4.0+ points | 4.0 or below |
| Response speed | 60 min | 10 min |
| Reply count | 3 times | 3 times |
| Social influence | Neutral | Recommended |
| Network environment | Good | Good |
| Price (CNY) | ¥0 | ¥200 |
| **Your choice** | □ | □ |
| **Choice Set ③ Which option would you prefer? Please tick your choice.** | | |
| Attributes | Option 1 | Option 2 |
| Number of articles | 2 articles | 2 articles |
| Physician rating | 4.0 or below | 4.0 or below |
| Response speed | 30 min | 10 min |
| Reply count | 8 times | 3 times |
| Social influence | Not recommended | Recommended |
| Network environment | Good | Good |
| Price (CNY) | ¥0 | ¥30 |
| **Your choice** | □ | □ |
| **Choice Set ④ Which option would you prefer? Please tick your choice.** | | |
| Attributes | Option 1 | Option 2 |
| Number of articles | 50 articles | 50 articles |
| Physician rating | 4.0 or below | 4.0+ points |
| Response speed | 60 min | 10 min |
| Reply count | 3 times | 8 times |
| Social influence | Not recommended | Recommended |
| Network environment | Moderate | Poor |
| Price (CNY) | ¥30 | ¥80 |
| **Your choice** | □ | □ |
| **Choice Set ⑤ Which option would you prefer? Please tick your choice.** | | |
| Attributes | Option 1 | Option 2 |
| Number of articles | 50 articles | 2 articles |
| Physician rating | 4.0+ points | 4.0+ points |
| Response speed | 30 min | 10 min |
| Reply count | 8 times | 3 times |
| Social influence | Not recommended | Not recommended |
| Network environment | Poor | Moderate |
| Price (CNY) | ¥0 | ¥80 |
| **Your choice** | □ | □ |
| **Choice Set ⑥ Which option would you prefer? Please tick your choice.** | | |
| Attributes | Option 1 | Option 2 |
| Number of articles | 50 articles | 2 articles |
| Physician rating | 4.0 or below | 4.0+ points |
| Response speed | 30 min | 10 min |
| Reply count | 8 times | 3 times |
| Social influence | Recommended | Not recommended |
| Network environment | Moderate | Good |
| Price (CNY) | ¥200 | ¥30 |
| **Your choice** | □ | □ |
| **Choice Set ⑦ Which option would you prefer? Please tick your choice.** | | |
| Attributes | Option 1 | Option 2 |
| Number of articles | 15 articles | 2 articles |
| Physician rating | 4.0 or below | 4.0+ points |
| Response speed | 10 min | 60 min |
| Reply count | 8 times | 8 times |
| Social influence | Not recommended | Recommended |
| Network environment | Poor | Moderate |
| Price (CNY) | ¥30 | ¥200 |
| **Your choice** | □ | □ |
| **Choice Set ⑧ Which option would you prefer? Please tick your choice.** | | |
| Attributes | Option 1 | Option 2 |
| Number of articles | 15 articles | 2 articles |
| Physician rating | 4.0 or below | 4.0+ points |
| Response speed | 10 min | 60 min |
| Reply count | 3 times | 3 times |
| Social influence | Not recommended | Not recommended |
| Network environment | Poor | Poor |
| Price (CNY) | ¥80 | ¥30 |
| **Your choice** | □ | □ |
| **Choice Set ⑨ Which option would you prefer? Please tick your choice.** | | |
| Attributes | Option 1 | Option 2 |
| Number of articles | 50 articles | 2 articles |
| Physician rating | 4.0+ points | 4.0 or below |
| Response speed | 10 min | 60 min |
| Reply count | 8 times | 3 times |
| Social influence | Recommended | Not recommended |
| Network environment | Good | Poor |
| Price (CNY) | ¥0 | ¥200 |
| **Your choice** | □ | □ |

# Additional file 2: Scales

**Mobile Health Literacy Scale**

Please indicate the extent to which you agree or disagree with the following statements.(① = Very inconsistent; ② = Inconsistent; ③ = Uncertain; ④ = Consistent; ⑤ = Very consistent)

| **Item** | **①** | **②** | **③** | **④** | **⑤** |
| --- | --- | --- | --- | --- | --- |
| I know which online platforms I can access for useful health and medical information. | ○ | ○ | ○ | ○ | ○ |
| I know how to use the health information I obtain online to help myself. | ○ | ○ | ○ | ○ | ○ |
| I have the cognitive ability to judge the quality of online health and medical information. | ○ | ○ | ○ | ○ | ○ |
| I feel confident in my ability to make health-related decisions using online information. | ○ | ○ | ○ | ○ | ○ |

**Trust**

Please indicate the extent to which you agree or disagree with the following statements based on your actual feelings. (① = Strongly disagree; ② = Disagree; ③ = Uncertain; ④ = Agree; ⑤ = Strongly agree.)

| **Item** | **①** | **②** | **③** | **④** | **⑤** |
| --- | --- | --- | --- | --- | --- |
| I believe that current healthcare knowledge platforms can protect my privacy and financial security. | ○ | ○ | ○ | ○ | ○ |
| I believe that the physicians and knowledge products on these platforms can help me address my health concerns. | ○ | ○ | ○ | ○ | ○ |
| I believe that the professionalism of physicians and content creators on healthcare knowledge platforms can be guaranteed. | ○ | ○ | ○ | ○ | ○ |

**Additional file 3: Model Comparison: Full Sample vs. Valid Sample**

**TABLE S1 Model Comparison: Full Sample vs. Valid Sample**

| Attribute | Full Sample (N=717) | | | Valid Sample (N=417) | | |
| --- | --- | --- | --- | --- | --- | --- |
|  | β | SE | p-value | β | SE | p-value |
| Article count (ref:2) | | | | | | |
| 15 articles | 0.225 | 0.129 | 0.082 | 0.663** | 0.227 | 0.003 |
| 50 articles | 0.622*** | 0.143 | <0.001 | 1.242*** | 0.248 | <0.001 |
| Physician rating (ref:≤4.0) | | | | | | |
| >4.0 points | -0.155 | 0.100 | 0.120 | -0.079 | 0.183 | 0.666 |
| Response speed (ref:60) | | | | | | |
| 30 min | 0.261 | 0.357 | 0.466 | 0.532 | 0.701 | 0.448 |
| 10 min | 0.608* | 0.297 | 0.041 | 1.214* | 0.618 | 0.049 |
| Reply count (ref:3) | | | | | | |
| 8 times | 0.533*** | 0.086 | <0.001 | 0.891*** | 0.154 | <0.001 |
| Social influence (ref:Not recommended) | | | | | | |
| Neutral | 0.844* | 0.372 | 0.023 | 1.507* | 0.726 | 0.038 |
| Recommended | 0.708*** | 0.114 | <0.001 | 0.966*** | 0.214 | <0.001 |
| Network environment (ref:Poor) | | | | | | |
| Moderate | 0.791*** | 0.223 | <0.001 | 1.382** | 0.45 | 0.002 |
| Good | 2.104*** | 0.462 | <0.001 | 3.33*** | 0.972 | 0.001 |
| Price (ref:0) | | | | | | |
| 30 CNY | -0.658*** | 0.165 | <0.001 | -1.127*** | 0.295 | <0.001 |
| 80 CNY | -1.445*** | 0.230 | <0.001 | -2.618*** | 0.472 | <0.001 |
| 200 CNY | -1.732*** | 0.219 | <0.001 | -3.391*** | 0.465 | <0.001 |

Note: *** p<0.001, ** p<0.01, * p<0.05

**Additional file 4: Linearity test for price**

**TABLE S2 Linearity test for price: quadratic specification**

| Attribute | Level | β | SE | p-value |
| --- | --- | --- | --- | --- |
| Article count | 15 articles (ref: 2) | 0.687*** | 0.189 | <0.001 |
|  | 50 articles (ref: 2) | 1.264*** | 0.223 | <0.001 |
| Physician rating | >4.0 points(ref: ≤4.0) | -0.091 | 0.172 | 0.595 |
| Response speed | 30 min(ref: 60) | 0.421 | 0.409 | 0.304 |
|  | 10 min(ref: 60) | 1.133** | 0.455 | 0.013 |
| Reply count | 8 times(ref: 3) | 0.882*** | 0.146 | <0.001 |
| Social influence | Neutral(ref: Not recommended) | 1.411** | 0.531 | 0.008 |
|  | Recommended(ref: Not recommended) | 0.942*** | 0.174 | <0.001 |
| Network environment | Moderate(ref: Poor) | 1.335*** | 0.377 | <0.001 |
|  | Good(ref: Poor) | 3.233*** | 0.825 | <0.001 |
| Price | — | -0.043*** | 0.008 | <0.001 |
| Price² | — | 0.000128*** | 0.000031 | <0.001 |

Note: ***p < 0.001, **p < 0.01, p < 0.05. WTP is not reported for this specification because the non-linear price term makes the standard WTP formula (ratio of attribute coefficient to price coefficient) invalid.

**TABLE S3 Linearity test for price: categorical specification**

| Attribute | Level | β | SE | p-value |
| --- | --- | --- | --- | --- |
| Article count | 15 articles (ref: 2) | 0.663** | 0.227 | 0.003 |
|  | 50 articles (ref: 2) | 1.242*** | 0.248 | <0.001 |
| Physician rating | >4.0 points(ref: ≤4.0) | -0.079 | 0.183 | 0.666 |
| Response speed | 30 min(ref: 60) | 0.532 | 0.701 | 0.448 |
|  | 10 min(ref: 60) | 1.214* | 0.618 | 0.049 |
|  | 3 times(ref) |  |  |  |
| Reply count | 8 times(ref: 3) | 0.891*** | 0.154 | <0.001 |
| Social influence | Neutral(ref: Not recommended) | 1.507* | 0.726 | 0.038 |
|  | Recommended(ref: Not recommended) | 0.966*** | 0.214 | <0.001 |
| Network environment | Moderate(ref: Poor) | 1.382** | 0.45 | 0.002 |
|  | Good(ref: Poor) | 3.33*** | 0.972 | 0.001 |
| Price | 30 CNY(ref: 0) | -1.127*** | 0.295 | <0.001 |
|  | 80 CNY(ref: 0) | -2.618*** | 0.472 | <0.001 |
|  | 200 CNY(ref: 0) | -3.391*** | 0.465 | <0.001 |

Note: ***p < 0.001, **p < 0.01, p < 0.05. WTP is not reported for this specification because the categorical price specification does not yield a single price coefficient for WTP calculation.

**Additional file 5: Moderating effects of mHealth literacy and trust**

**TABLE S4 Moderating effects of mHealth literacy and trust**

| **Attribute** | **Level** | **β** | **SE** | **Exp(β)** |
| --- | --- | --- | --- | --- |
| Article count | 15 articles (ref: 2) | 0.760*** | 0.152 | 2.139 |
|  | 50 articles (ref: 2) | 0.910*** | 0.153 | 2.485 |
| Physician rating | >4.0 points(ref: ≤4.0) | 0.167 | 0.117 | 1.182 |
| Response speed | 30 min (ref: 60) | -0.635*** | 0.191 | 0.530 |
|  | 10 min (ref: 60) | -0.194 | 0.145 | 0.824 |
| Reply count | 8 times (ref: 3) | 0.466*** | 0.084 | 1.593 |
| Social influence | Neutral (ref: Not recommended) | -0.101 | 0.184 | 0.903 |
|  | Recommended (ref: Not recommended) | 0.751*** | 0.109 | 2.119 |
| Network environment | Moderate (ref: Poor) | 0.146 | 0.114 | 1.157 |
|  | Good (ref: Poor) | 0.586** | 0.203 | 1.797 |
| Price | — | -0.011*** | 0.001 | 0.989 |
| Trust × Social influence | Neutral (ref: Not recommended) | -0.053 | 0.149 | 0.948 |
|  | Recommended (ref: Not recommended) | -0.309** | 0.098 | 0.734 |
| Trust × Network environment | Moderate (ref: Poor) | -0.027 | 0.104 | 0.973 |
|  | Good (ref: Poor) | -0.328* | 0.167 | 0.720 |
| mHealth literacy × Article count | 15 articles (ref: 2) | -0.086 | 0.110 | 0.917 |
|  | 50 articles (ref: 2) | 0.153 | 0.111 | 1.165 |
| mHealth literacy × Price | — | 0.002** | 0.001 | 1.002 |

Note: ***p < 0.001, **p < 0.01,*p < 0.05. SE = standard error; Exp(β) = exponentiated coefficient. Reference levels are indicated by “ref.”

**Additional file 6: Preferences and WTP by gender**

**TABLE S5 Preferences and WTP by gender**

| **Attribute** | **Level** | **Male (N=120)** | | **Female (N=297)** | |
| --- | --- | --- | --- | --- | --- |
|  |  | β | WTP (CNY) | β | WTP (CNY) |
| Article count | 15 articles (ref: 2) | 0.700** | 99.99 | 0.757*** | 54.09 |
|  | 50 articles (ref: 2) | 0.856*** | 122.33 | 0.925*** | 66.10 |
| Physician rating | >4.0 points(ref: ≤4.0) | 0.312 | 44.63 | 0.058 | 4.11 |
| Response speed | 30 min (ref: 60) | -0.841** | -120.09 | -0.415 | -29.67 |
|  | 10 min (ref: 60) | -0.301 | -42.94 | -0.090 | -6.46 |
| Reply count | 8 times (ref: 3) | 0.518*** | 73.99 | 0.410*** | 29.31 |
| Social influence | Neutral (ref: Not recommended) | -0.332 | -47.39 | 0.116 | 8.30 |
|  | Recommended (ref: Not recommended) | 0.515*** | 73.52 | 0.906*** | 64.74 |
| Network environment | Moderate (ref: Poor) | 0.120 | 17.14 | 0.199 | 14.21 |
|  | Good (ref: Poor) | 0.165 | 23.53 | 0.934*** | 66.74 |
| Price | — | -0.007*** | — | -0.014*** | — |
| mHealth literacy × Price | — | 0.002** | — | 0.001 | — |
| Trust × Social influence | Neutral (ref: Not recommended) | -0.053 | — | -0.036 | — |
|  | Recommended (ref: Not recommended) | -0.310* | — | -0.260* | — |
| Trust × Network environment | Moderate (ref: Poor) | -0.101 | — | -0.009 | — |
|  | Good (ref: Poor) | -0.367 | — | -0.305 | — |

Note: **p < 0.01, ***p < 0.001,*p < 0.05. Price coefficients are shown for reference; WTP for price is not applicable as it serves as the denominator in WTP calculations.

**Additional file 7: Preferences and WTP by age group**

**TABLE S6 Preferences and WTP by age group**

| **Attribute** | **Level** | **18–25 (N=175)** | | **26–40 (N=124)** | | **≥41 (N=118)** | |
| --- | --- | --- | --- | --- | --- | --- | --- |
|  |  | β | WTP (CNY) | β | WTP (CNY) | β | WTP (CNY) |
| Article count | 15 articles (ref: 2) | 1.045*** | 80.38 | 0.824* | 58.87 | 0.380 | 47.52 |
|  | 50 articles (ref: 2) | 1.159*** | 89.19 | 1.250*** | 89.29 | 0.399 | 49.90 |
| Physician rating | >4.0 points(ref: ≤4.0) | 0.376 | 28.90 | 0.199 | 14.20 | -0.103 | -12.82 |
| Response speed | 30 min (ref: 60) | -0.810* | -62.27 | -0.736 | -52.56 | -0.252 | -31.46 |
|  | 10 min (ref: 60) | -0.173 | -13.29 | -0.297 | -21.18 | -0.018 | -2.23 |
| Reply count | 8 times (ref: 3) | 0.574*** | 44.13 | 0.583** | 41.65 | 0.302* | 37.74 |
| Social influence | Neutral (ref: Not recommended) | -0.130 | -10.02 | 0.020 | 1.42 | -0.005 | -0.56 |
|  | Recommended (ref: Not recommended) | 0.717*** | 55.18 | 0.651** | 46.50 | 0.869*** | 108.61 |
| Network environment | Moderate (ref: Poor) | 0.183 | 14.10 | 0.196 | 14.03 | 0.131 | 16.41 |
|  | Good (ref: Poor) | 0.611 | 47.02 | 0.900* | 64.31 | 0.557 | 69.60 |
| Price | — | -0.013*** | — | -0.014*** | — | -0.008*** | — |
| mHealth literacy × Price | — | -0.001 | — | 0.006** | — | 0.002** | — |
| Trust × Social influence | Neutral (ref: Not recommended) | -0.269 | — | 0.137 | — | 0.013 | — |
|  | Recommended (ref: Not recommended) | -0.219 | — | -0.647** | — | -0.222 | — |
| Trust × Network environment | Moderate (ref: Poor) | -0.069 | — | -0.096 | — | -0.095 | — |
|  | Good (ref: Poor) | -0.257 | — | -0.713 | — | -0.367 | — |

Note: ***p < 0.001, **p < 0.01,*p < 0.05. Price coefficients are shown for reference; WTP for price is not applicable as it serves as the denominator in WTP calculations.

**Additional file 8: Preferences and WTP by OMC experience**

**TABLE S7 Preferences and WTP by OMC experience**

| **Attribute** | **Level** | **Experienced users (N=357)** | | **First-time users (N=60)** | |
| --- | --- | --- | --- | --- | --- |
|  |  | β | WTP (CNY) | β | WTP (CNY) |
| Article count | 15 articles (ref: 2) | 0.863*** | 66.35 | 0.387 | 55.30 |
|  | 50 articles (ref: 2) | 1.054*** | 81.11 | 0.413 | 59.06 |
| Physician rating | >4.0 points(ref: ≤4.0) | 0.266 | 20.48 | -0.185 | -26.39 |
| Response speed | 30 min (ref: 60) | -0.658** | -50.62 | -0.429 | -61.22 |
|  | 10 min (ref: 60) | -0.112 | -8.62 | -0.417 | -59.62 |
| Reply count | 8 times (ref: 3) | 0.528*** | 40.59 | 0.216 | 30.87 |
| Social influence | Neutral (ref: Not recommended) | -0.105 | -8.07 | 0.076 | 10.88 |
|  | Recommended (ref: Not recommended) | 0.819*** | 62.99 | 0.474* | 67.66 |
| Network environment | Moderate (ref: Poor) | 0.201 | 15.43 | 0.006 | 0.86 |
|  | Good (ref: Poor) | 0.581* | 44.71 | 0.786 | 112.23 |
| Price | — | -0.013*** | — | -0.007*** | — |
| mHealth literacy × Price | — | 0.002** | — | 0.001 | — |
| Trust × Social influence | Neutral (ref: Not recommended) | -0.057 | — | -0.010 | — |
|  | Recommended (ref: Not recommended) | -0.320** | — | -0.337 | — |
| Trust × Network environment | Moderate (ref: Poor) | -0.086 | — | 0.136 | — |
|  | Good (ref: Poor) | -0.430* | — | 0.129 | — |

Note: ***p < 0.001, **p < 0.01,*p < 0.05. Price coefficients are shown for reference; WTP for price is not applicable as it serves as the denominator in WTP calculations.

**Additional file 9: Preferences and WTP by disease context**

**TABLE S8 Preferences and WTP by disease context**

| **Attribute** | **Level** | **Mild condition (N=185)** | | **Severe condition (N=232)** | |
| --- | --- | --- | --- | --- | --- |
|  |  | β | WTP (CNY) | β | WTP (CNY) |
| Article count | 15 articles (ref: 2) | 0.738*** | 56.78 | 0.672*** | 74.69 |
|  | 50 articles (ref: 2) | 0.731*** | 56.27 | 0.945*** | 105.01 |
| Physician rating | >4.0 points(ref: ≤4.0) | 0.122 | 9.39 | 0.209 | 23.28 |
| Response speed | 30 min (ref: 60) | -1.252*** | -96.27 | -0.052 | -5.79 |
|  | 10 min (ref: 60) | -0.722*** | -55.57 | 0.297 | 33.05 |
| Reply count | 8 times (ref: 3) | 0.409*** | 31.49 | 0.492*** | 54.70 |
| Social influence | Neutral (ref: Not recommended) | -0.878*** | -67.52 | 0.512* | 56.88 |
|  | Recommended (ref: Not recommended) | 0.497*** | 38.22 | 0.994*** | 110.41 |
| Network environment | Moderate (ref: Poor) | -0.155 | -11.92 | 0.405** | 45.02 |
|  | Good (ref: Poor) | -0.150 | -11.55 | 1.137*** | 126.39 |
| Price | — | -0.013*** | — | -0.009*** | — |
| mHealth literacy × Price | — | 0.003** | — | 0.001 | — |
| Trust × Social influence | Neutral (ref: Not recommended) | -0.118 | — | -0.028 | — |
|  | Recommended (ref: Not recommended) | -0.281* | — | -0.180 | — |
| Trust × Network environment | Moderate (ref: Poor) | -0.004 | — | -0.081 | — |
|  | Good (ref: Poor) | -0.283 | — | -0.417* | — |

Note: ***p < 0.001, **p < 0.01,*p < 0.05. Price coefficients are shown for reference; WTP for price is not applicable as it serves as the denominator in WTP calculations.

**Additional file 10: Latent class logit models**

**TABLE S9 Two-class latent class logit model results — 3 core attributes**

| **Attribute and level** | **Class 1 (price-sensitive)** | | **Class 2 (price-insensitive)** | |
| --- | --- | --- | --- | --- |
|  | **Coefficient (SE)** | **p value** | **Coefficient (SE)** | **p value** |
| Social influence (ref: Not recommended) | — | — | — | — |
| Neutral | -0.356** (0.147) | 0.016 | 0.070 (0.199) | 0.724 |
| Recommended | 1.360*** (0.124) | 0.000 | 0.559*** (0.117) | 0.000 |
| Article count (ref: 2) | — | — | — | — |
| 15 articles | 0.230** (0.097) | 0.018 | 0.241** (0.109) | 0.028 |
| 50 articles | 0.291** (0.114) | 0.010 | 0.049 (0.125) | 0.697 |
| Price (ref: 0) | — | — | — | — |
| 30 CNY | -0.181 (0.135) | 0.181 | 0.217 (0.178) | 0.223 |
| 80 CNY | -1.056*** (0.167) | 0.000 | 0.421* (0.158) | 0.068 |
| 200 CNY | -2.792*** (0.258) | 0.000 | 0.300 (0.259) | 0.247 |
| Class share (EM) | 0.627 |  | 0.373 |  |
| Class size, n (%) | 276 (66.19%) |  | 141 (33.81%) |  |
| Average posterior probability | 0.630 |  | 0.370 |  |
| Log-likelihood | -2035.044 |  |  |  |
| AIC | 4100.088 |  |  |  |
| BIC | 4160.584 |  |  |  |

Note: *** p < 0.01, ** p < 0.05, * p < 0.10. Coefficients were estimated using maximum likelihood; standard errors in parentheses. Class labels were assigned based on price sensitivity. The 3-attribute model excludes reply count.

**TABLE S10 Two-class latent class logit model results — 4 core attributes**

| **Attribute and level** | **Class 1 (price-sensitive)** | | **Class 2 (price-insensitive)** | |
| --- | --- | --- | --- | --- |
|  | **Coefficient (SE)** | **p value** | **Coefficient (SE)** | **p value** |
| Social influence (ref: Not recommended) | — | — | — | — |
| Neutral | 0.007(0.224) | 0.973 | -0.491***(0.177) | 0.006 |
| Recommended | 2.118***(0.325) | 0.000 | -0.213(0.186) | 0.252 |
| Reply count (ref: 3) | — | — | — | — |
| 8 times | -0.167(0.121) | 0.171 | 0.234***(0.085) | 0.006 |
| Article count (ref: 2) | — | — | — | — |
| 15 articles | 0.172*(0.102) | 0.092 | 0.308**(0.112) | 0.028 |
| 50 articles | 0.224*(0.119) | 0.059 | 0.064(0.115) | 0.576 |
| Price (ref: 0) | — | — | — | — |
| 30 CNY | -0.166(0.183) | 0.365 | 0.285(0.184) | 0.122 |
| 80 CNY | -0.471***(0.149) | 0.002 | -0.339**(0.130) | 0.009 |
| 200 CNY | -2.316***(0.333) | 0.000 | -0.711***(0.189) | 0.000 |
| Class share (EM) | 0.577 |  | 0.423 |  |
| Class size, n (%) | 262（62.83%） |  | 155（37.17） |  |
| Average posterior probability | 0.583 |  | 0.417 |  |
| Log-likelihood | -2038.167 |  |  |  |
| AIC | 4110.334 |  |  |  |
| BIC | 4178.896 |  |  |  |

Note: *** p < 0.01, ** p < 0.05, * p < 0.10. Coefficients were estimated using maximum likelihood; standard errors in parentheses. Class labels were assigned based on price sensitivity.

**TABLE S11 Agreement in class assignment between 4-attribute and 3-attribute models**

|  | **3-attribute Class 1** | **3-attribute Class 2** | **Total** |
| --- | --- | --- | --- |
| 4-attribute Class 1 | 190 | 72 | 262 |
| 4-attribute Class 2 | 86 | 69 | 155 |
| Total | 276 | 141 | 417 |

Note: Diagonal agreement = 259 respondents (62.11%); cross-classification = 158 respondents (37.89%)
